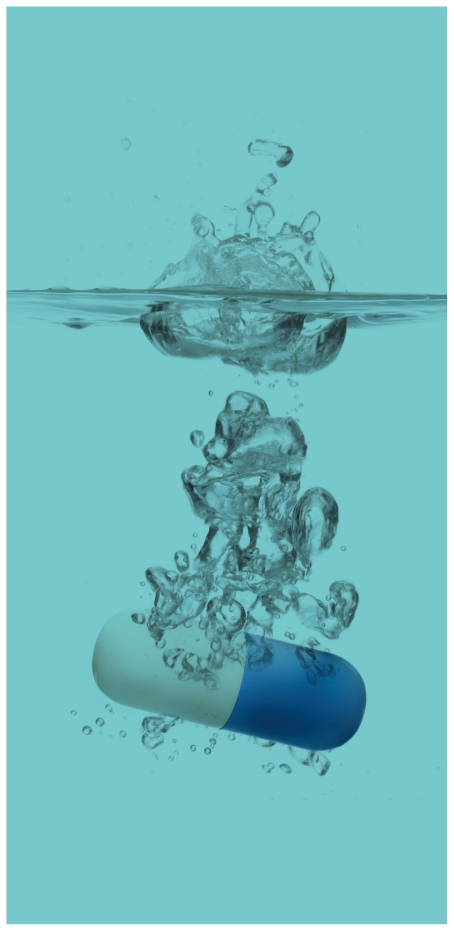# Drugs in the Environment: Do Pharmaceutical Take-Back Programs Make a Difference?

**DOI:** 10.1289/ehp.118-a210

**Published:** 2010-05

**Authors:** Naomi Lubick

**Affiliations:** **Naomi Lubick** is a freelance science writer based in Zürich, Switzerland, and Folsom, CA. She has written for *Environmental Science & Technology*, *Nature*, and *Earth*

The state of Maine experimented with drugs last year. The state had already tested several methods for collecting unused pharmaceuticals, with varying degrees of success. After tracking surprisingly high concentrations of pharmaceuticals in landfill leachate —raising the potential for eventual ground and surface water contamination—the state decided to pursue a new tool to keep drugs out of the waste stream. Maine wanted to establish statewide collection programs, mandated by legislation and paid for by manufacturers, that would intercept unwanted pharmaceutical products before they got to the trash.

Although the state legislation bogged down earlier this year, other states have introduced bills similar to Maine’s, with some success. Meanwhile, Europe and Canada have had systems for pharmaceutical take-back programs in place for a decade or so. At the same time, an increasing number of reports from across the world have tracked active pharmaceutical ingredients (APIs) in surface waters and even tap water, leading environmental scientists and water utilities to look for ways to limit the amount of drugs entering the environment.

The bulk of human pharmaceuticals found in waterways most likely got there by way of sewage. Taking unused pharmaceuticals out of landfills may make only a small difference in the concentrations of APIs found in water, say critics and supporters alike of such programs. But take-back programs may help prevent leftover pharmaceuticals from being misused. For that reason and others, utilities and local governments are moving forward with a variety of pharmaceutical take-back efforts in the absence of regulations—or data indicating such programs actually work.

## Digging into the Trash

The most damning evidence yet of human drugs’ impacts on wildlife comes from studies of fish. A study by Karen Kidd et al., in the 22 May 2007 issue of *Proceedings of the National Academy of Sciences*, showed the collapse of a population of fish in an isolated lake spiked with relatively high levels of the synthetic estrogen 17α-ethinylestradiol. Other studies on aquatic populations in a waste-impacted stream in Boulder, Colorado, showed reproductive effects from estrogenic wastewater effluent, according to Alan M. Vajda et al., writing in the 1 May 2008 issue of *Environmental Science & Technology*.

Aside from any environmental implications, discarded pharmaceuticals pose the threat of misuse. “People will go to great lengths to reclaim a drug,” says Christian Daughton of the U.S. Environmental Protection Agency. He cites reports of “pee labs,” where a dealer might reclaim methamphetamine from a user’s urine and reconstitute it for resale. But it’s not only drug users who go through the garbage for pharmaceuticals, says Ann Pistell, an environmental specialist at the Maine Department of Environmental Protection (DEP): “It’s accidental poisonings by children, pets, or wildlife who pluck them out.”

In January 2010, the Maine DEP measured the concentrations of drugs in samples of leachate collected at three landfills, selected because they were receiving only household waste and not biosolids that might contain human-excreted drugs. DEP scientists were surprised to find what could amount to yearly leachate emissions of hundreds of pounds of APIs from over-the-counter and prescription drugs. “The fact that we found pharmaceuticals wasn’t a huge surprise, but the high levels were,” says Pistell. The pain reliever acetaminophen, for example, was present in samples from one landfill at concentrations of 117,000 ng/L, the highest level of any drug measured in the study.

The prescription antibiotic ciprofloxacin was present at concentrations of 269 ng/L, and lab tests even found cocaine—at 57 ng/L—in one landfill, according to the DEP’s unpublished findings. Other drugs found in all three landfills included low concentrations of estrone (from hormone replacement therapy), albuterol (an asthma drug), and the antibiotic penicillin in the range of tens to hundreds parts per billion.

The findings led the DEP to decide that disposing of unused pharmaceutical products in landfills—the current recommendation of U.S. industry and government to consumers wanting to clean out their medicine cabinets—is not a prudent or sustainable method of disposal.

According to the SMARxT Disposal™ partnership, however, landfills are fine for disposal of pharmaceuticals. This partnership of the U.S. Fish and Wildlife Service, the American Pharmacists Association, and the Pharmaceutical Research and Manufacturers of America recommends that medications be crushed and/or dissolved, mixed with kitty litter or other unappealing material (to discourage consumption), then enclosed in a container or sealable baggie before disposal in the trash. The U.S. Office of National Drug Control Policy, in its 2009 “Proper Disposal of Prescription Drugs” factsheet, agrees with this advice where take-back programs are not available. Unused controlled drugs collected by law enforcement typically are incinerated—considered the most effective way to destroy APIs—through licensed medical waste collectors.

Susan Boehme, who studies contaminated coastal sediments with the Illinois–Indiana Sea Grant program, says life-cycle analyses of drug disposal methods are not yet complete. As someone who spends a lot of time helping communities set up and operate pharmaceutical take-back programs, Boehme says she cautions stakeholders that the impacts of a local take-back program on pollution prevention often will be unclear and that such programs are “definitely a precautionary approach.”

Few studies have been conducted on pharmaceuticals in landfills and leachate, says Dana Kolpin of the U.S. Geological Survey, lead author of a landmark paper published 15 March 2002 in *Environmental Science & Technology* that showed the widespread presence of pharmaceuticals in U.S. surface waters. Kolpin and his colleagues previously examined pharmaceuticals present in groundwater leachate plumes, and the team currently is attempting to organize a national survey of landfill leachate to better understand the levels of pharmaceuticals that may be present. Many landfill operations actually collect leachate for further treatment at a wastewater treatment plant, which may make this a slower pathway for drugs to travel into the environment, Kolpin says, “but it’s still a potential pathway.”

Kolpin adds, “At some point, somebody has to look and find the mass balance, so to speak.” How much of the pharmaceuticals in the environment, whether excreted or unused, come from residential waste versus hospitals versus farms? What sources are the biggest contributors? Not many answers exist to these questions at the moment, most researchers say.

## Filling in the Blanks

Currently, says Ilene Ruhoy of Touro University Nevada, take-back programs are “not standardized in any way.” Data collection from people turning in drugs may differ from event to event, as might methods used to classify them by type and measure the amount of drugs collected—for example, whether that amount reflects the mass of the medication in its packaging, the mass of the complete formulated product (APIs plus excipients), or the mass of just the APIs. These differences make extrapolating data from an event to figure out its impact “really complicated,” she says, without even adding the complexities of the potential ecological effects of each drug.

Duane Huggett of the University of North Texas hopes to fill in some of the blanks on the exact benefits of take-back programs. For the City of Denton’s collection in late April 2010, Huggett and his colleagues established a protocol for collecting drugs while logging statistically valid data for later evaluation. They hope to repeat this pilot program at future events across the United States.

As more states roll out take-back legislation and programs, the country could end up with 50 different state programs, Huggett says, and standardization, at least in data collection, would certainly help in assessing the impacts of these programs, if not their establishment and implementation. Moreover, without regulation, some of these programs may not even be legal, according to Jen Jackson of East Bay Municipal Utility District, the public utility serving San Francisco’s East Bay. For example, she says, until California set up its own guidance for water utilities and pharmacies to collect unused pharmaceuticals, the state’s many take-back programs were operating in a legal gray zone.

That’s in large part because pharmaceutical take-back programs are subject to the same rules that are meant to keep controlled substances from reentering the supply chain either legally or illegally: under the federal Controlled Substances Act, the U.S. Drug Enforcement Administration requires controlled substances to be turned in to the proper law enforcement officials. Any collection program must be carefully monitored by law enforcement, Jackson says, so nothing is diverted from a collection box, for example. The additional monitoring needed for these events can increase event expenses.

Jackson says pharmacies must be very careful to involve as few hands as possible in take-back programs. For instance, in California the public can deposit unused pharmaceuticals in one-way bins with two-key systems. Collections of full bins might take place with a licensed medical waste hauler and a pharmacist present as witnesses to ensure drugs in high demand on the street (such as the neurostimulant Ritalin and the analgesic Vicodin) are not diverted from their path to destruction.

LD 821, the Maine bill introduced in March 2009 by Representative Anne Perry, would have required industry to assist in establishing take-back programs for unused pharmaceuticals in that state. The bill called for manufacturers to demonstrate to the Maine DEP that they were taking part in or running their own take-back programs, with proper disposal of their products through hazardous waste incinerators. The bill also called for pharmacies to provide prepaid envelopes so customers could mail unused pharmaceuticals back to the manufacturer. The bill passed Maine’s House of Representatives by a wide margin but was tabled in the state Senate in March 2010.

In contrast to the United States, Europe has widespread standardized take-back programs. In the 2010 report *Pharmaceuticals in the Environment: Results of an EEA Workshop*, the European Environment Agency (EEA) stated most countries there collect unused drugs separately from household waste, usually at pharmacies (a handful also have separate collection sites alongside pharmacies). But even in Europe, not all unused pharmaceuticals are diverted from the waste stream. A survey from Germany’s Management Strategies for Pharmaceutical Residues in Drinking Water (*start*) research program showed that consumers discarded 23% of liquid pharmaceuticals prescribed and 7% of tablets. While some went into household trash, the proportion that went down the drain amounted to 364 tons of APIs flushed away every year. Only about a third of the population surveyed by the *start* program reported always returning their drugs to a pharmacy.

## End of the Line

Daughton and Ruhoy have developed a methodology that could be used to quantify unused pharmaceuticals that end up in the waste stream in the United States, using coroners’ records and other data sources. In the 15 December 2007 issue of *Science of the Total Environment*, they note that medical investigators from coroners’ offices routinely search decedents’ homes for drugs in case they played a role in a death, and the coroner often maintains detailed records of the pharmaceuticals found and their method of disposal. Creating a unified network of coroners’ databases from around the country could yield valuable insight into the types and amounts of pharmaceuticals consumers tend to accumulate.

Researchers have also examined how to diminish environmental impacts of pharmaceuticals using the principles of green chemistry: Ruhoy says more manufacturers have found ways to use less water or solvents and thereby lessen the environmental impacts of pharmaceutical production. Still, few have rolled out products that might easily biodegrade in the environment. In the May 2003 issue of *EHP*, Daughton suggested expanding the use of “optically pure chiral drugs” to reduce by half—or sometimes more—the amount of API required in a medication. A chiral (or “handed”) molecule may have mirror-image configurations that are not quite identical; one form may be more effective by fitting into certain receptors, whereas another may be ineffective or even harmful because of its different form. Focusing on the optimal configuration of a molecule selects for materials that can be used more efficiently by the human body while cutting down on pharmaceutical bulk.

Another possibility for reducing the impact of APIs in the environment involves advising medical professionals about drugs that are less environmentally harmful. In Sweden the pharmaceutical industry has assisted the government in putting together a database of the possible environmental effects of various medications. A patient could select a less environmentally persistent painkiller, for example, by avoiding off-label use of the anticonvulsant carbamazepine. Europe is currently examining how to expand this Swedish Environmental Classification of Pharmaceuticals database to the international level.

Daughton points out that reduced usage, lower dosages for personalized medicines tailored to an individual’s genome, and other approaches could cut down on human excretion of drugs to the environment—and the need to dispose of unused pharmaceuticals—while perhaps achieving better health care outcomes. “One of the downsides of focusing on drug disposal is that it serves to distract from the issues that could potentially have much more impact on the occurrence of APIs in the environment,” he comments. “This is especially true given that we don’t even know the relative contributions of APIs in the environment that result from disposal versus intended usage.”

Although the question of whether pharmaceutical take-back programs make a difference does not yet have a clear answer, Kolpin remains optimistic about the possibilities for keeping pharmaceuticals out of the environment, observing that “more and more people [are] working on the issue . . . and providing results that advance the science.” He says that although it may be unrealistic to eliminate every contaminant from waste, perhaps researchers and regulators could focus on the “bad actors,” those compounds known to be the most common or most harmful. Some argue that consumers could have the most impact on the amount of pharmaceuticals in the environment, for example in choosing to buy fewer or “greener” pharmaceuticals.

Meanwhile, Maine’s LD 821 bill may yet see another day. Pistell says the bill will be reintroduced in January 2011 by a new sponsor, and that it will go to a natural resources committee—which is more familiar with product stewardship issues—instead of one on public health. The state already has refined the bill after hearing legislators’ concerns, according to Pistell, who explains, “lots of bills take several years to get through.” She adds, “Those who have greatest influence over a product—usually manufacturers—certainly should have a role in dealing responsibly with a product at the end of its life.”

## Save a Flush

Only certain drugs approved by the Food and Drug Administration should be flushed down the toilet or drain. These include drugs deemed to be “especially harmful to a child, pet, or anyone else if taken accidentally,” according to the agency’s “Information for Consumers (Drugs)” webpage.

Once APIs reach the bacteria that clean up wastewater in treatment plants, drugs may degrade into daughter compounds that may be more or less toxic than the parent drug, or they may even return to their original forms. Some will flush out with treated effluent into streams, and some will be captured in biosolids—the sludge left over after water treatment—that might end up incinerated, spread over agricultural lands, or placed in landfills. A study by Dana Kolpin and colleagues in the 15 March 2008 issue of *Environmental Science & Technology* found that earthworms from fields where biosolids had been spread as fertilizer had measurable amounts of pharmaceuticals in their bodies.

Pharmaceuticals flushed into septic systems may pose even more of a threat to waterways than those put into municiple systems, according to some of the few studies on the topic. Conversely, study findings published in the February 2010 issue of *Environmental Toxicology and Chemistry* suggest that septic tanks may remove organic contaminants as effectively as wastewater treatment plants, although there are no data yet on specific drugs.

## For More Information

National Programs and Institutes (a list of take-back programs and other resources in various states) Teleosis Institute

http://www.teleosis.org/gpp-national.php

The Drug Take-Back Network News (recent news about drug take-back issues) Product Stewardship Institute

http://www.takebacknetwork.com/news_t.php

Swedish Environmental Classification of Pharmaceuticals Swedish Association of the Pharmaceutical Industry

http://www.fass.se/LIF/miljo_splash/index_en.jsp

## Figures and Tables

**Figure:**
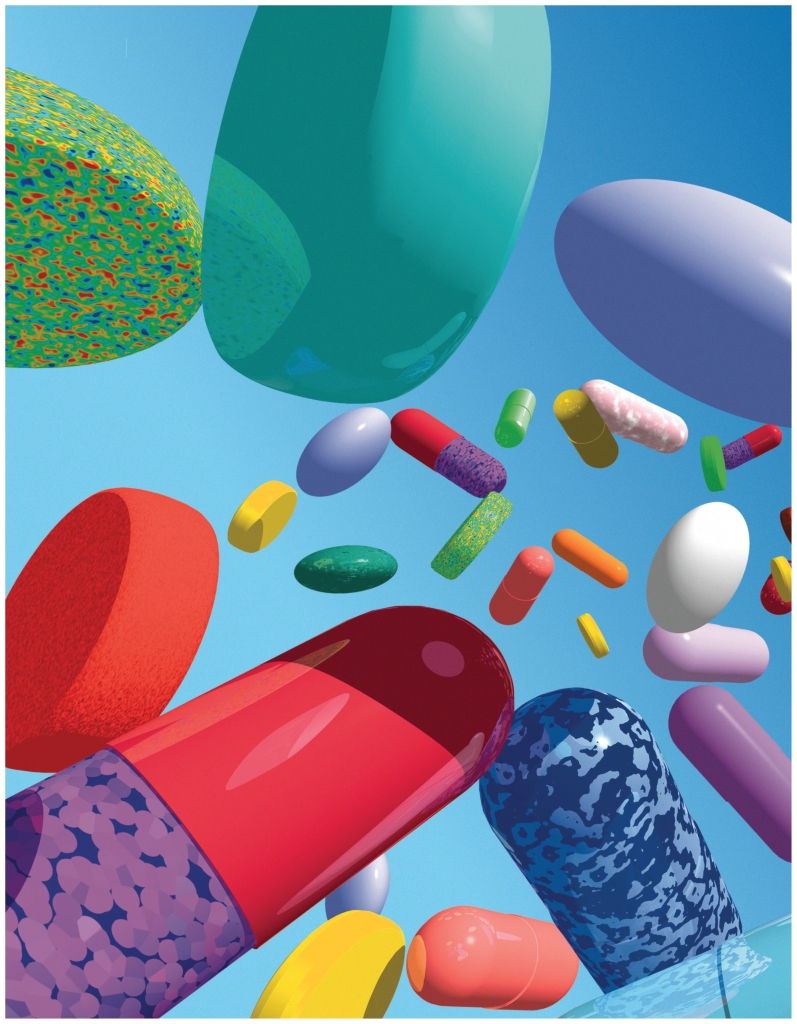


**Figure:**